# Coadministration of Monophosphoryl Lipid and Curcumin Modulates Neuroprotective Effects in LPS Stimulated Rat Primary Microglial Cells

**DOI:** 10.1155/omcl/9422312

**Published:** 2024-11-28

**Authors:** Maryam Hooshmand, Ahmad Asoodeh

**Affiliations:** Department of Chemistry, Faculty of Science, Ferdowsi University of Mashhad, Mashhad, Iran

**Keywords:** curcumin, in vitro model, microglia, monophosphoryl lipid A, neuroinflammation, neuroprotection, primary cell culture

## Abstract

Lipopolysaccharide (LPS)-induced activation of microglia triggers the release of neuroinflammatory molecules, contributing to the progression of neurodegenerative diseases. Targeting these neuroinflammatory molecules could serve as a potential therapeutic strategy. Given the evidence supporting the immune-boosting properties of curcumin (Curc) and the protective effects of monophosphoryl lipid A (MPL) in the central nervous system (CNS) related to Alzheimer's disease (AD), this study aimed to assess the anti-inflammatory effects of these compounds on primary rat microglial cells, which are crucial in the response to neuroinflammation. This in vitro study investigated the effects of Curc, MPL, and their coadministration (Curc + MPL) on inflammatory cytokine levels in activated microglial cells. Primary microglial cells were isolated from 1-day-old rats and treated with various concentrations of Curc, MPL, and Curc + MPL prior to LPS stimulation. Cell viability was assessed using the MTT assay, followed by the Griess assay to evaluate nitric oxide (NO) production. The levels of inflammatory cytokines interleukin-1*β* (IL-1*β*), tumor necrosis factor-alpha (TNF-*α*), and interleukin-6 (IL-6), as well as the gene expression of inducible NO synthase (iNOS) and cyclooxygenase-2 (COX-2), were analyzed via real-time PCR. Additionally, enzyme-linked immunosorbent assay (ELISA) was employed to quantify the protein levels of IL-1*β*, TNF-*α*, and IL-6. Our findings demonstrate that Curc and MPL possess antineuroinflammatory properties in LPS-stimulated microglial cells. Notably, the coadministration of Curc and MPL (Curc + MPL) significantly inhibited the production of pro-inflammatory cytokines IL-1*β*, TNF-*α*, and IL-6. Furthermore, Curc + MPL suppressed the expression of iNOS and COX-2. These results strongly suggest that Curc + MPL is a promising neuroprotective agent for the treatment of neurodegenerative disorders by mitigating neuroinflammatory responses.

## 1. Introduction

The prevalence of neurodegenerative diseases in individuals aged 65 and above is a pressing public health concern. These conditions entail the buildup of misfolded proteins in the central nervous system (CNS), triggering inflammation and consequent neuronal loss [1]. With the aging population, the financial impact of age-related disorders, especially neurodegenerative illnesses, is poised to escalate, necessitating effective preventive or therapeutic strategies [2].

Neuroinflammation, a common feature of many neurodegenerative diseases, denotes an inflammatory response within the CNS, often resulting from various pathological factors like infection, trauma, or toxins [3]. This inflammatory process involves the release of pro-inflammatory molecules by innate immune cells in the CNS, potentially leading to tissue damage and cell death [4]. This cell demise, through apoptosis or necroptosis, underlies neurodegenerative pathologies such as Parkinson's disease (PD) and Alzheimer's disease (AD), which together account for a substantial portion of diagnosed cases [5–7].

Microglial cells, integral components of the CNS's innate immune system, play a pivotal role in maintaining CNS balance and responding to inflammatory triggers [8]. Normally, they release inflammatory substances to combat foreign elements and infections. However, when activated, they lose the equilibrium between free radicals and antioxidant defenses, resulting in increased production of various inflammatory mediators [9, 10]. Overactivated microglia are implicated in conditions like AD, PD, traumatic brain injury, or aging [11]. Therefore, compounds with antioxidant properties might mitigate neuroinflammation by curbing oxidative stress, potentially offering therapeutic benefits for neurodegenerative diseases.

Nitric oxide (NO) is essential for synaptic transmission and brain plasticity, particularly in the cortex and hippocampus. However, excessive NO levels can induce cellular stress and contribute to nitrosative and oxidative stress, potentially disrupting synaptic plasticity and accelerating neurodegeneration. The production and activation of inducible NO synthase (iNOS) are driven by inflammatory agents, including interleukin-1, interferon-gamma (IFN-*γ*), and nuclear factor kappa-light-chain-enhancer of activated B cells (NF-*κ*B) in astrocytes and microglia, leading to elevated NO levels [12]. Cyclooxygenase-2 (COX-2), initially identified as a key player in the acute inflammatory response, is rapidly induced by various inflammatory stimuli. Expression of COX-2 and the resulting microglial activation can cause secondary neuronal damage and alter the surrounding cellular environment [13].

Microglia, the resident immune cells of the CNS, are primed to respond to inflammation and predominantly express various cytokines, such as interleukin-6 (IL-6), interleukin-1*β* (IL-1*β*), and tumor necrosis factor-alpha (TNF-*α*). TNF-*α* has traditionally been considered a potent inflammatory mediator that contributes to tissue degeneration in several disease conditions. However, recent evidence indicates that TNF-*α* may also have a role in regenerative processes [14]. IL-6, commonly associated with inflammatory and pathological conditions, is also crucial in maintaining normal brain function [15]. IL-1*β* is a pro-inflammatory cytokine that plays a vital role in regulating the host's innate immune response. It is essential for managing inflammation against microbial infections and facilitating tissue repair mechanisms. Disruptions in homeostasis trigger the production of IL-1*β*, enabling it to exert its inflammatory effects [16]. In this investigation, we delve into the synergistic potential of curcumin (Curc), a natural polyphenol derived from turmeric, and monophosphoryl lipid A (MPL), a Toll-like receptor 4 (TLR4) agonist, to address CNS-induced microglial inflammation. Curc is renowned for its robust anti-inflammatory and antioxidant properties, modulating antioxidant enzyme activity and influencing pathways associated with NF-*κ*B signaling. Similarly, MPL, derived from Salmonella Minnesota, exhibits notable anti-inflammatory properties and is utilized as an adjuvant in various vaccines due to its exceptional safety profile and efficacy.

A wealth of prior research has highlighted the individual anti-inflammatory attributes of Curc and MPL. Curc has demonstrated the ability to suppress lipopolysaccharide (LPS)-induced COX-2 expression and inhibit various inflammatory mediators such as TNF-*α*, PGE2, NO, iNOS, and COX-2 [17]. Moreover, it impedes LTA-induced MAPK phosphorylation and NF-*κ*B translocation in microglial cells [18].

In this regard, MPL administration has shown protective effects against brain injury and epilepsy in rats [19], reducing seizure susceptibility and ameliorating depressive-like behaviors induced by chronic stress [20]. MPL has also exhibited efficacy in enhancing memory performance in models of AD [21] and hippocampal ischemia [22], suggesting promising neuroprotective benefits.

Given the compelling individual effects of Curc and MPL, our study aims to unveil the combined impact of these agents on CNS-induced microglial inflammation. This exploration into the novel synergistic potential of Curc and MPL may yield valuable insights into prospective therapeutic strategies for neuroinflammatory conditions.

## 2. Materials and Methods

**Figure 1:**
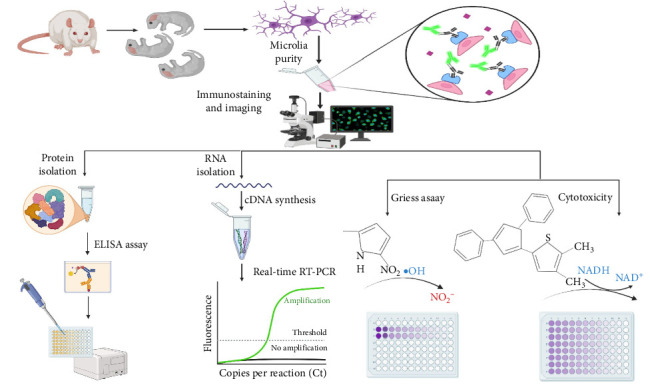
The primary use of microglia is to assess the anti-inflammatory properties of Curc + MPL. The schematic diagram illustrates the process of obtaining microglia from newborn rats (1-day old), which are then suspended in DMEM with 20% FBS to support microglial growth. After a minimum of 14 days, microglia are harvested by agitation. The collected microglia are cultured and examined using fluorescent microscopy to assess their purity. Additionally, the viability and nitric oxide production (measured using Griess assay) of inflamed microglia pretreated with Curc, MPL, and Curc + MPL are evaluated. Finally, changes in inflammatory factors of inflamed microglia pretreated with Curc, MPL, and Curc + MPL were analyzed using real-time RT-PCR and ELISA. ELISA, enzyme-linked immunosorbent assay; FBS, fetal bovine serum; MPL, monophosphoryl lipid A.

This research was conducted to evaluate the anti-inflammatory capacity of Curc, MPL, and their combination on rat microglial cells, the immune players of CNS in response to neuroinflammation (Figure 1).

### 2.1. Microglia Cell Culture

**Figure 2:**
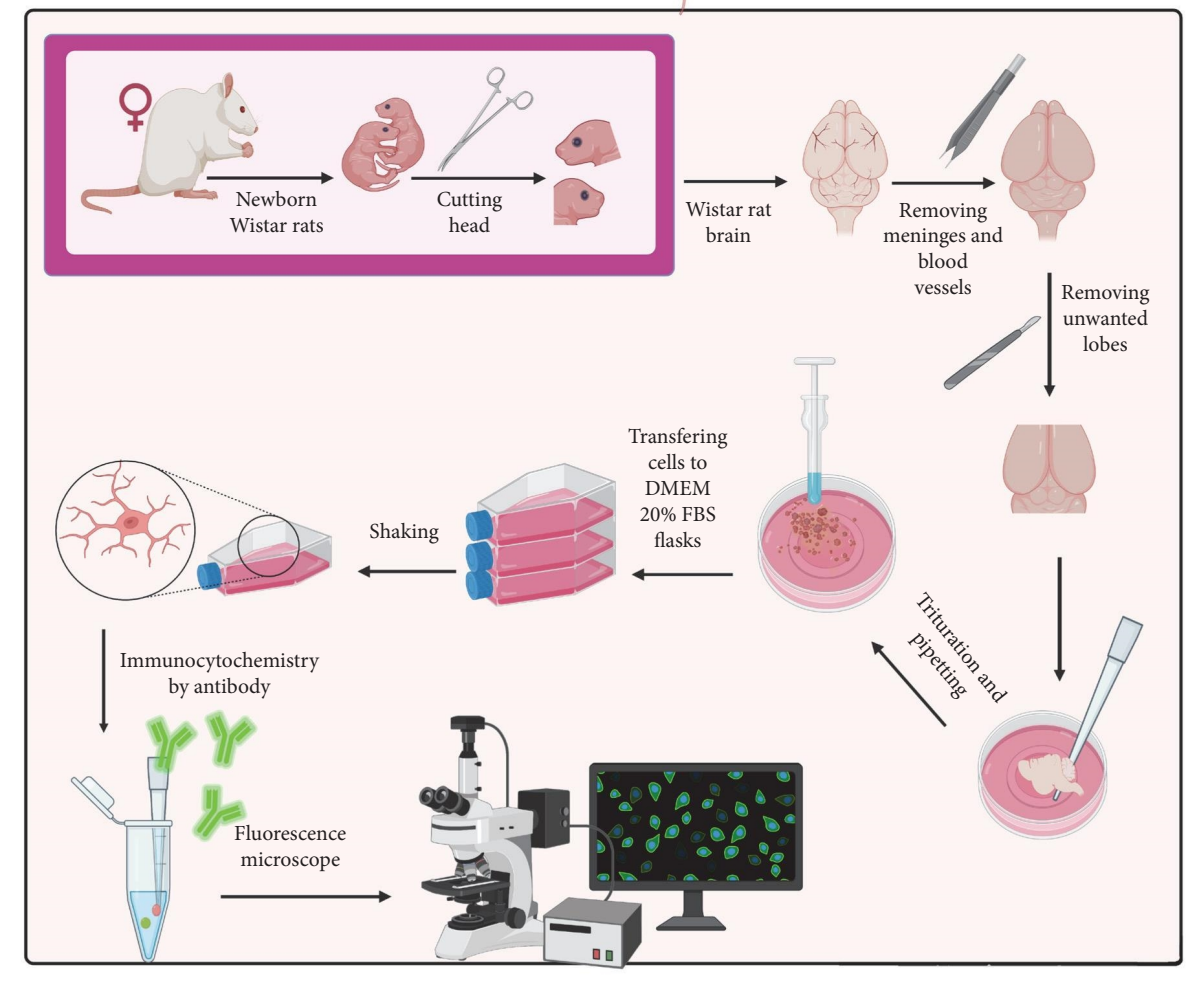
Schematic figure depicting the obtaining primary microglia culture from 1-day-old newborn Wistar rats. After removing meninges and blood vessels, the olfactory lobes and cerebellum are separated. All remaining are mechanically disrupted and transferred to T-25 flasks containing high-glucose DMEM + 20% FBS culture. After 14 days, primary microglia were extracted by shaking. The purity of microglia was determined by Iba-1 antibodies and the fluorescence emission, detected by a fluorescence microscope (Nikon, Japan). FBS, fetal bovine serum.

Microglial cells were isolated from the whole brains of Wistar rats at postnatal days 0–1, with an average weight of 1.4 g [23]. The isolation process involved the complete removal of the olfactory lobes, cerebellum, and meninges with blood vessels from the brain. The remaining brain tissue was mechanically dissociated in DMEM, and the floating cells were transferred to T-25 flasks containing high-glucose DMEM supplemented with 20% fetal bovine serum (FBS) in a humidified atmosphere of 95% air and 5% CO_2_. After washing to remove nonadherent cells, the cultures were maintained in DMEM with 10% FBS, with medium changes twice a week. Following 14 days of culture, when the mixed glial cultures reached 80% confluency, the cultures were gently shaken for 2–5 min to detach microglial cells. The suspended microglia were collected for further experiments. The protocol for isolating primary microglia from the brains of newborn rats and assessing their purity is detailed in Figure 2.

### 2.2. Drugs

MPL (Sigma-Aldrich, USA) was dissolved in phosphate-buffered saline (PBS) in a stock of 500 µg/ml. The 1 µg/ml was applied in microglia primary cell culture; the dose was selected based on the effective doses used in Alzheimer's and stroke studies [21, 22]. Curc (Sigma-Aldrich, USA) was dissolved in DMSO and added to the studied concentrations.

### 2.3. Immunocytochemistry (ICC)

The purity of our microglial cultures was determined by rabbit anti-rat/mouse Iba-1 (Abcam, Cambridge, UK) as microglia-specific markers and goat antirabbit IgG (Abcam, Cambridge, UK) as the secondary antibody. Briefly, microglial cells fixed in 4% paraformaldehyde were suspended in PBST (0.3% Triton x-100 in PBS) for 20 min, followed by adding the blocking reagent containing 0.3% Triton X-100 in PBS, 10% Normal Donkey Serum (Abcam, Cambridge, UK) and BSA. Afterward, cells were incubated with rabbit anti-rat Iba1 antibody (1:250), with Hoechst (Invitrogen H3569, 1:5000) for 45 min in the dark. Finally, after incubation of cells in goat anti-rabbit IgG (1:400) for 45 min, primary microglia cells were ascended with Fluoromount-G (Thermo Fisher, Massachusetts, USA) mounting medium using a fluorescence microscope. GFAPCy3 (Sigma, USA, 1:500) was used as a negative control to detect contamination.

### 2.4. MTT Assay for Cell Viability

To determine whether Curc, MPL, and Curc + MPL influence the viability of inflamed primary microglia cells after treatment with Curc, the MTT (3-(4,5dimethylthiazol-2-yl)-2,5-diphenyltetrazolium bromide) (Sigma, MO, USA) assay was performed. About 1.5 × 10^4^ primary microglial cells (per well) were seeded in 96 well plates. The wells were devoted to triplicates. After 24 h, by removing the supernatant, cells were pretreated by different concentrations of Curc (10, 20, 50, 70, 90 µg/ml), MPL (1 µg/ml), and Curc + MPL (11, 21 µg/ml), 30 min before activation by LPS (1 µg/ml, Sigma, Santa Clara, CA, USA). The cell viability of inflamed pretreated microglia by different doses of Curc was determined after 24 h using a spectrophotometer at 580 nm.

### 2.5. NO Determination

NO production by the inflamed primary microglial cells was assessed by evaluating the nitrite concentrations in the culture medium using Griess reagent [24]. Briefly, 2 × 10^4^ cells/ml primary microglia cells were seeded in a 96-well plate (SPL Life Sciences Co, Korea). Cells were stimulated with 1 µg/ml LPS. After 24 h, the supernatant was collected and mixed with an identical volume of Griess reagent, and the final nitrite concentration was observed at 540 nm, using a standard curve of sodium nitrite, which was performed serially diluted solutions (0–150 mM).

### 2.6. RT-PCR and Quantitative Real-Time PCR (qPCR)

The total RNA was isolated from primary microglial cells using Trizol's (Gibco, Grand Island, NY, USA) manufacturer instructions. The purity of total RNA was determined by spectrophotometric optical density measurement (ratio of 260–280 nm). This ratio (OD_260_/OD_280_) was used to provide an estimate of the purity of the nucleic acid [25]. Consequently, the ratio of all prepared RNA samples ranged between the purity range (1.7–2.0). About 1 µg amount of RNA was reversely transcribed into complementary DNA (cDNA) using a cDNA synthesis Kit (iNtRON Biotechnology, Korea) according to the manufacturer's protocol. The synthesized cDNA was used as a template in PCR reaction using specific primers for iNOS2, IL-1*β*, IL-6, TNF-*α*, and Cox-2 (Table 1). GAPDH was utilized as an internal control. PCR products were separated by 1.5% agarose gel electrophoresis and visualized with SYBR DNA gel dye (Invitrogen, USA).

For qPCR studies, quantitative changes in mRNA levels were estimated by real-time PCR using RealQ Plus 2x Master Mix Green High (Ampliqon, Denmark). The synthesized cDNA (1 μl) was used as a template in PCR reaction. The amplification cycles were performed with an initial denaturation for 5 min hold at 95°C, followed by 35 cycles of denaturation at 95°C (30 s), annealing at 59°C (30 s), and extension at 72°C (30 s). A single fluorescence reading was recorded at each extension step. The relative mRNA expression and fold changes in the expression of iNOS, IL-1*β*, IL-6, TNF-*α*, and Cox-2 were calculated using the mean cycle threshold (ΔCt) values from quadruplicate measurements after normalizing to the level of GADPH gene expression as an internal control using the means of the comparative Ct with 2^−*ΔΔCT*^ equation by REST software. The resulting amplicons were examined by melting peaks and 1% agarose gel electrophoresis [26]. Furthermore, primer sequences are available in Table 1.

### 2.7. Measurement of IL-1*β*, IL-6, and TNF-*α* Levels by Enzyme-Linked Immunosorbent Assay (ELISA)

Primary isolated microglia cells were plated overnight in 24-well plates at a density of 3 × 10^5^ cells per plate. The cells were pretreated studied concentrations of Curc, MPL, and Curc + MPL were added to the medium 30 min prior to LPS induction for 24 h. Supernatants were then centrifuged at 10,000 *g* for 10 min, and supernatants were collected and determined the total protein concentration. The supernatant was stored at −70°C. The levels of IL-1*β*, IL-6, and TNF-*α* proteins in the culture medium were determined by a mouse ELISA kit (R&D Systems, Minneapolis, MN, USA) according to manufacturers' instructions. The cytokines concentrations were expressed as picograms per milligram of protein.

### 2.8. Statistical Analysis

Data were expressed as mean ± SD and processed by commercially available software Graph Pad Prism 9.4. version. The normal distribution of data was analyzed by the Shapiro–Wilk's test. The data were normally distributed (*p*  > 0.05). Therefore, cell viability and inflammatory factors fold change were analyzed by parametric tests two-way ANOVA followed by Tukey's test. NO production and the level of cytokines density were analyzed by one-way ANOVA followed by Tukey's test. Statistical differences for all tests were considered significant at *p*  < 0.05 value.

## 3. Results

### 3.1. Examination of Microglia Purity

**Figure 3:**
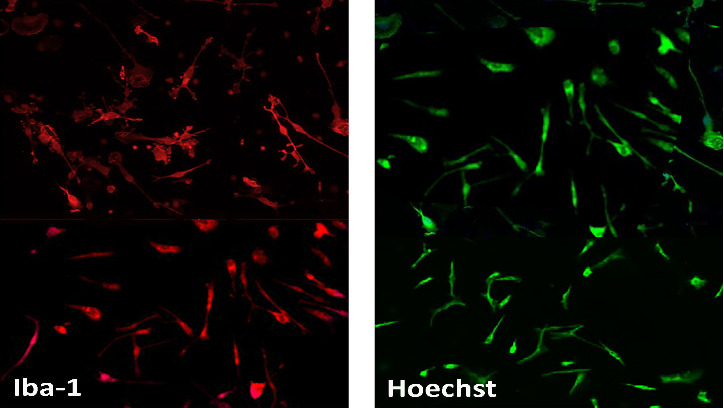
Characterization of purified microglia after 24 h by ICC. Microscopy images = 20× magnification. ICC, immunocytochemistry.

The purity of microglial cells, isolated from 1-day post-natal Wistar rats was 98% determined by ICC assay (Figure 3).

### 3.2. Cell Viability of LPS-Stimulated Microglia With Curc

**Figure 4:**
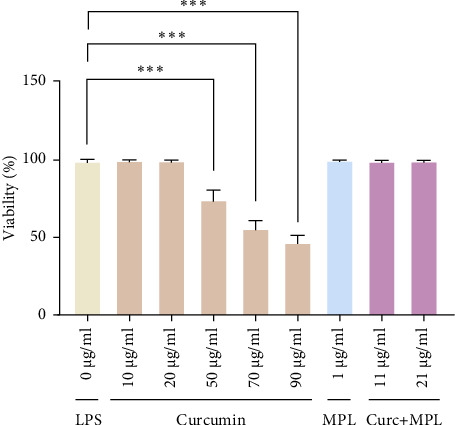
Viability assay of microglia cells inflamed by LPS as control (0 µg/ml) and pretreated microglial cells by 10, 20, 50,70, 90 µg/ml concentrations of Curc, 1 µg/ml MPL and 11, 21 µg/ml concentrations of Curc + MPL after 24 h. The data are expressed as mean ± SEM. Statistical analyses were carried out by using two-way ANOVA followed by Tukey's test with *⁣*^*∗∗∗*^*p*  < 0.001 showing toxic effect at 50,70, 90 µg/ml and no significance at 10, 20 µg/ml concentrations of Curc, and applied concentrations of MPL and Curc + MPL against the LPS-stimulated control. Curc, curcumin; LPS, lipopolysaccharide; MPL, monophosphoryl lipid A.

The primary microglial cells were treated with 10, 20, 50, 70, and 90 µg/ml of Curc concentrations, 1 µg/ml MPL, and 11, 21 µg/ml concentrations of Curc + MPL prior to the LPS application. Curc showed a toxic effect at 50, 70, and 90 µg/ml and no significant toxicity comparable to control at 10, 20 µg/ml doses, which were selected in the subsequent experiments. MPL and Curc + MPL applied concentrations had no significant toxicity (Figure 4).

### 3.3. NO Production in the Inflamed Microglia

**Figure 5:**
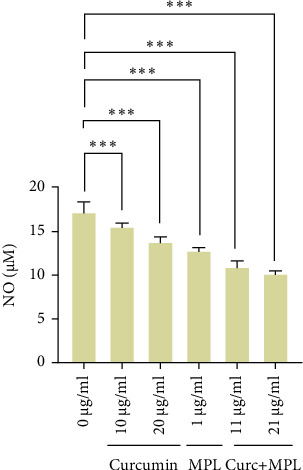
Nitric oxide evaluation in microglia cells inflamed by LPS as control (0 µg/ml) and pretreated inflamed microglial cells by studied amounts of Curc, MPL, and Curc + MPL after 24 h. The data are expressed as mean ± SEM. Statistical analyses were carried out using one-way ANOVA followed by Tukey's test with *⁣*^*∗∗∗*^*p*  < 0.001 and *⁣*^*∗∗∗*^*p*  < 0.001, indicating statistically significant differences in comparison to the LPS-stimulated control. Curc, curcumin; LPS, lipopolysaccharide; MPL, monophosphoryl lipid A.

**Figure 6:**
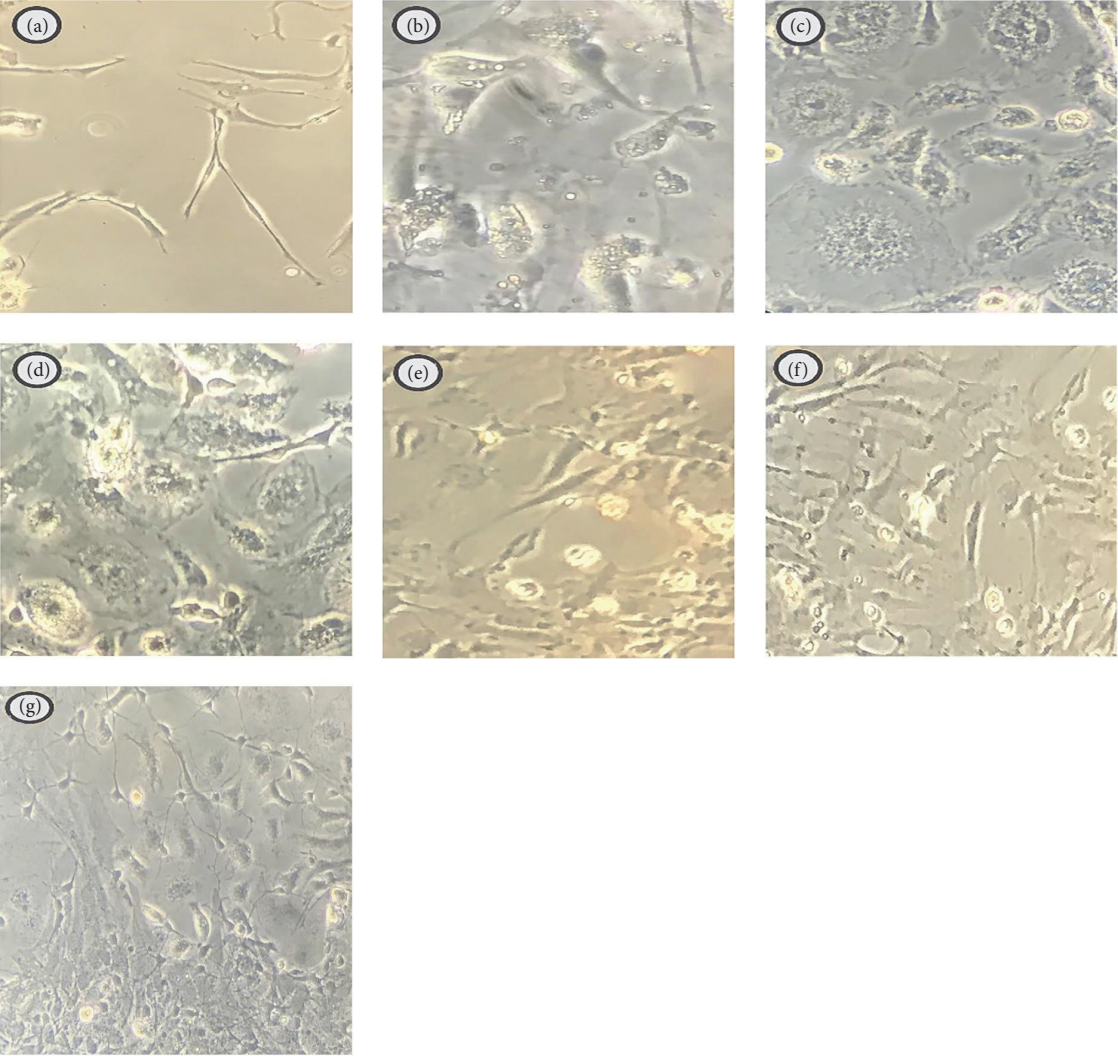
Illustrates the (a) morphology of rat-purified normal microglia cells after 24 h and (b) purified microglia cells activated by LPS as well as purified microglia cells pretreated with Curc (c) 10 µg/ml, (d) 20 µg/ml, MPL (e) 1 µg/ml and (f) 11 µg/ml, (g) 21 µg/ml Curc + MPL, 30 min prior to LPS addition, for 24 h. The figures were observed and captured by an inverted microscope scope × 20. Curc, curcumin; LPS, lipopolysaccharide; MPL, monophosphoryl lipid A.

NO production was significantly suppressed upon using the effective doses of Curc (10, 20 µg/ml), MPL (1 µg/ml), and Curc + MPL (11, 21 µg/ml) shown in Figure 5. All in all, accompanied by observed data of morphological changes of microglia, it is confirmed that the cells were better ramified upon the application of selected doses of Curc, MPL, and Curc + MPL (Figure 6). The inflamed cells mostly had ameboid morphology, while the pretreated inflamed cells' morphotypes shifted mostly from ameboid to ramified.

### 3.4. iNOS, COX-2, IL-1*β*, IL-6, and TNF-*α* mRNA Expression

**Figure 7:**
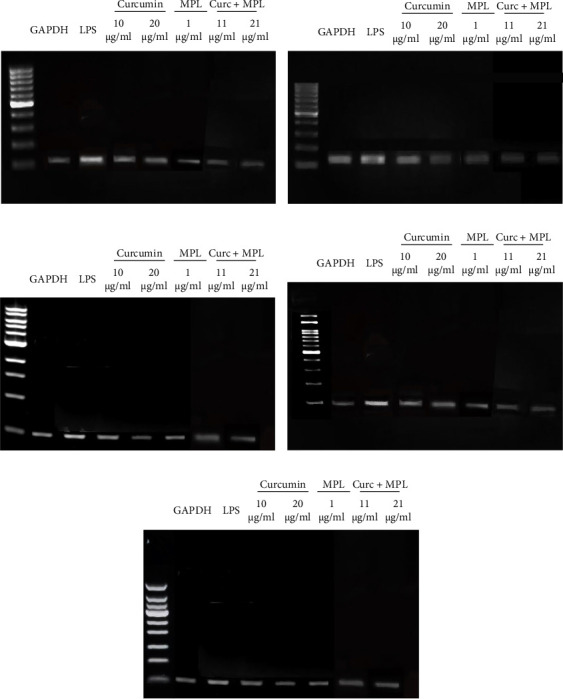
Presents The effects of Curc on gene expressions of (a) *iNOS*, (b) *Cox-2*, (c) *IL-1β*, (d) *TNF-α*, and (e) *IL-6* in pretreated induced microglia, respectively. Cox-2, cyclooxygenase-2; Curc, curcumin; IL-1*β*, interleukin-1*β*; IL-6, interleukin-6; iNOS, inducible NO synthase; TNF-*α*, tumor necrosis factor-alpha.

**Figure 8:**
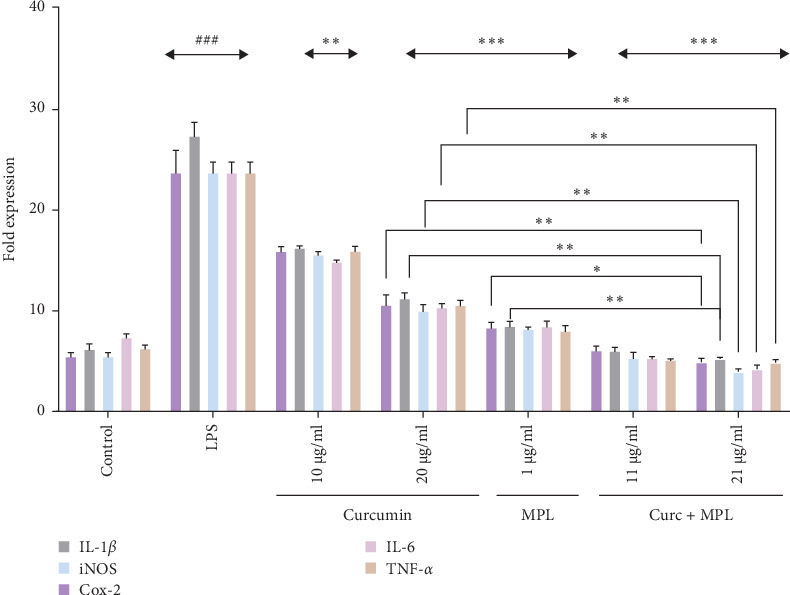
Effect of Curc, MPL and Curc + MPL on *iNOS*, *Cox-2*, *IL-6*, *TNF-α*, and *IL-1β* mRNA expression in treated induced microglia cells. Results were quantified by real-time PCR using cDNA synthesized from isolated RNA. *GAPDH* was used as a housekeeping gene for normalization. Untreated microglia were used as a reference. Analyzing the figures disclosed significant downregulation of the pro-inflammatory genes in induced pretreated microglia upon the application of 10, 20 µg/ml of Curc, 1 µg/ml of MPL, and 11, 21 µg/ml concentrations of Curc + MPL. The relative gene expression level of each tested gene was calculated using the 2^−*ΔΔCT*^ Pffafl's method and is presented in log_2_ form. Statistical analyses were carried out by using one-way ANOVA test with *⁣*^*∗*^*p*  < 0.05, *⁣*^*∗∗*^*p*  < 0.01, and *⁣*^*∗∗∗*^*p* < 0.001, indicating statistically significant differences against the LPS-stimulated positive control. ^###^ Indicates *p* < 0.001 difference between control and LPS groups. Cox-2, cyclooxygenase-2; Curc, curcumin; IL-1*β*, interleukin-1*β*; IL-6, interleukin-6; iNOS, inducible NO synthase; LPS, lipopolysaccharide; MPL, monophosphoryl lipid A; TNF-*α*, tumor necrosis factor-alpha.

We investigated the mRNA expression levels of key pro-inflammatory factors, including iNOS, COX-2, IL-1*β*, IL-6, and TNF-*α*, in LPS-induced inflamed microglia treated with varying concentrations of Curc, MPL, and Curc + MPL using real-time PCR (Figure 7). The findings demonstrated a dose-dependent reduction in the expression of these pro-inflammatory genes upon pretreatment with the studied compounds. Inflamed microglia respond to LPS stimulation by upregulating the expression of pro-inflammatory cytokines and enzymes, TNF-*α*, IL-6, IL-1*β*, iNOS, and COX-2. However, pretreatment with Curc (10 and 20 µg/ml), MPL (1 µg/ml), and Curc + MPL (11 and 21 µg/ml) significantly attenuated the expression of these genes in a dose-dependent manner. Specifically, treatment with 10 µg/ml of Curc resulted in a notable two-fold decrease in mRNA levels compared to untreated inflamed microglia. Increasing the concentration of Curc to 20 µg/ml and combining it with MPL (Curc + MPL) at studied concentrations (11 and 21 µg/ml) led to more substantial reductions, with mRNA levels decreasing ~4–4.5-fold across the evaluated pro-inflammatory factors.  Figure 8 visually represents the dose-dependent reductions in mRNA expression observed in the treated inflamed microglia cells.

### 3.5. TNF-*α* and IL-6 and IL-1*β* Expression by ELISA

**Figure 9:**
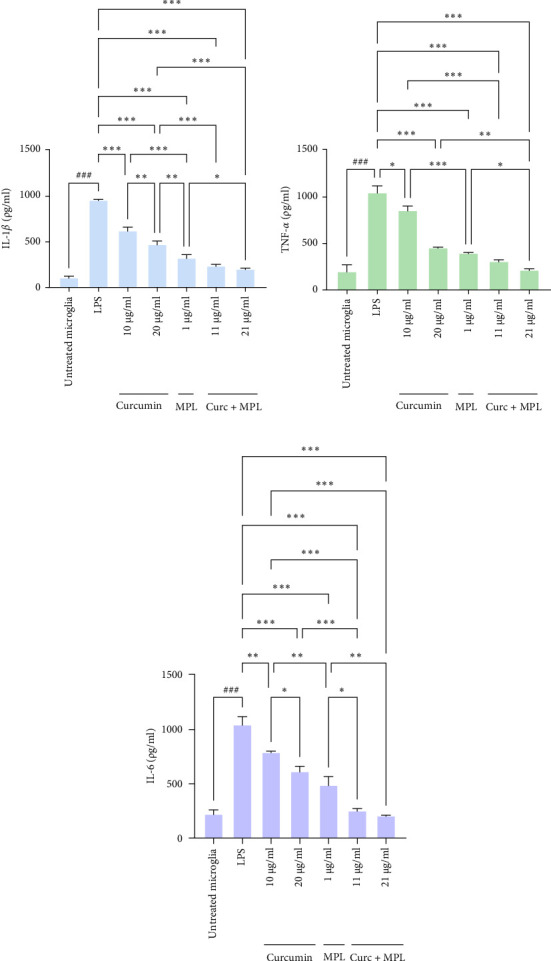
Effects of Curc, MPL, and Curc + MPL on the levels of (a) IL-1*β*, (b) TNF-*α*, and (c) IL-6 in the presence of LPS; the cells were pretreated with different concentrations of compounds for 30 min and then incubated with LPS for 24 h. The levels (pg/ml) of IL-1*β*, TNF-*α*, and IL-6 were determined in the presence of LPS. Data statistically analyzed by one-way ANOVA test with ^###^*p*  < 0.0001 versus control; *⁣*^*∗*^*p*  < 0.05, *⁣*^*∗∗*^*p*  < 0.01, and *⁣*^*∗∗∗*^*p*  < 0.001, indicating statistically significant differences against the LPS-stimulated positive control. Curc, curcumin; IL-1*β*, interleukin-1*β*; IL-6, interleukin-6; LPS, lipopolysaccharide; MPL, monophosphoryl lipid A; TNF-*α*, tumor necrosis factor-alpha.

The cells were pretreated with Curc (10, 20 µg/ml), MPL (1 µg/ml), and Curc + MPL (11, 21 µg/ml) for 30 min, and then LPS was added. After 24 h, TNF-*α*, IL-1*β*, and IL-6 were measured by using ELISA. In comparison with control, LPS significantly increased IL-1*β* (*p*  < 0.001), TNF-*α* (*p*  < 0.001), and IL-6 (*p*  < 0.001) in the supernatant. As shown in Figure 9a–c, Curc significantly reduced IL-1*β* (*p*  < 0.001), TNF-*α* (*p*  < 0.05), and IL-6 (*p*  < 0.01) in 10 µg/ml and *p*  < 0.001 in 20 µg/ml. On the other hand, MPL declined the studied factors significantly (*p*  < 0.001). The Curc + MPL in 11 µg/ml significantly decreased the IL-1*β*, TNF-*α* (*p*  < 0.001), and IL-6 (*p*  < 0.001). It also decreased the factors to *p*  < 0.0001 in 21 µg/ml in the presence of LPS. On the other hand, IL-1*β* was reduced by *p*  < 0.001 in 21 µg/ml Curc + MPL treatment in comparison with 20 µg/ml Curc treatment and *p*  < 0.05 against MPL treatment. Furthermore, the 21 µg/ml of Curc + MPL reduced TNF-*α* by *p*  < 0.05 against MPL and Curc. Finally, IL-6 levels in 21 µg/ml Curc + MPL, declined significantly against MPL (*p*  < 0.05) and 20 µg/ml Curc (*p*  < 0.01).

## 4. Discussion

Neuroinflammation stands as a pivotal aspect of various neurological disorders, including AD, PD, acute traumatic brain injury, and infectious neuropathology [27, 28]. While microglia, the key immune cells in the CNS, play a neuroprotective role by releasing anti-inflammatory agents, an imbalance in this process triggers microglial overactivation, leading to excessive production of NO and pro-inflammatory cytokines, contributing to various neuroinflammatory conditions [29]. In LPS-induced microglia, increased levels of pro-inflammatory factors are observed based on a number of observational studies [30–32].

Ensuring the safety of any drug, natural or synthetic, is crucial. In our investigation, we assessed the cytotoxic effects of Curc, MPL, and Curc + MPL on LPS-induced primary microglia cells, confirming minimal to null toxicity at selected concentrations. Related to our study, a combination of 20 µg/ml Curc and 100 ng/ml LPS had no cytotoxic effects on microglia cells [33]. It is reported that Curc had no effect on microglia viability [34, 35].

Additionally, Curc, MPL, and Curc + MPL effectively suppressed NO production in inflamed primary microglia cells, as evidenced by both NO evaluation data and observed morphological changes. Activated microglia undergo significant changes in morphology, from a small cell body with long branches (ramified) to a round shape with short branches, which is known as ameboid [36]. Immunofluorescence detection of Iba-1 revealed that LPS stimulation decreased the length of cell branches and increased cell body diameter as compared to the control group. These changes were reversed by Curc, MPL, and Curc + MPL treatment, which is in consistence with previous studies that showed pre-retreatment with Curc ameliorated the induced morphological changes in BV2 cells [37]. MPL, being a modified form of LPS, has a significant impact on induced microglia. It appears to induce a notable change in microglia morphology towards a more ramified (less ameboid) state. This suggests that MPL can modulate microglia activation and morphology, potentially influencing the inflammatory response triggered by LPS. A previous study demonstrated that MPL stimulates cytoskeletal remodeling, albeit to a lower extent compared to LPS [38]. This parallel supports the notion that MPL has a moderate impact on microglial cytoskeleton dynamics and activity and emphasized MPL's distinct and potentially more subtle influence on microglial behavior compared to LPS.

Moreover, our study revealed that Curc inhibited the expression of iNOS and COX-2 mRNA, key contributors to the generation of cytotoxic pro-inflammatory factors by activated microglia. This inhibition correlated with decreased production of downstream pro-inflammatory cytokines such as IL-1*β*, IL-6, and TNF-*α* [33, 34], which are typically elevated during inflammation mediated by LPS via the MAPK signaling pathway in microglia cells [39, 40]. Recent research also suggests that Curc downregulates NF-*κ*B protein levels, further reducing pro-inflammatory mediators [41]. MPL and Curc + MPL also decreased the levels of pro-inflammatory cytokines, in which Curc + MPL was more profound, which is referred to MPL's action promoting a shift toward a more regulatory or anti-inflammatory phenotype in immune cells, which could be beneficial in attenuating excessive inflammation [42, 43]. Curc, a natural polyphenol derived from turmeric, has been shown to inhibit inflammatory pathways, including NF-*κ*B activation, leading to reduced cytokine production and inflammatory gene expression in microglia. Similarly, MPL, a TLR4 agonist derived from bacterial LPS, can induce immune tolerance and shift immune responses towards an anti-inflammatory phenotype [44, 45]. The combination of Curc and MPL may have synergistic or additive effects in modulating microglial inflammation, although further optimization of dosing and treatment protocols may be necessary to achieve significant reductions in inflammatory markers. The lack of statistical significance with Curc + MPL compared to individual treatments could be attributed to several factors, including experimental variability, suboptimal concentrations, or the need for longer treatment durations to observe robust effects.

Future studies could explore the underlying molecular mechanisms by which Curc and MPL exert their anti-inflammatory effects in microglia, such as examining their impact on specific signaling pathways and transcription factors involved in neuroinflammation. Additionally, investigating the long-term effects and potential neuroprotective benefits of Curc and MPL treatment in neurodegenerative disease models would provide valuable insights into their therapeutic potential for mitigating neuroinflammatory processes.

In light of these findings, our study firmly establishes Curc and MPL anti-inflammatory effects on LPS-stimulated primary microglia cells, evidenced by reduced expression of pro-inflammatory genes and cytokine release from inflamed microglia cells.

## 5. Conclusion

To put it in a nut shell, our study highlights the promising anti-inflammatory properties of Curc and MPL in reducing microglial activation and inflammatory responses. Considering the synergic anti-inflammatory effects of the combination of MPL and Curc, it is anticipated that they can be involved in the preparation of future neuroprotective medicines. In this regard, further researches is warranted to optimize treatment strategies and explore their translational applications in neuroinflammatory disorders such as neurodegeneration diseases.

## Figures and Tables

**Table 1:** Sequences of primers used for RT-PCR and qPCR.

Name of genes	Forward primer sequences (5′→3′)	Reverse primer sequences (5′→3′)
GAPDH	5′TGCACCACCAACTGCTTA3′	5′GGATGCAGGGATGATGTTC3′
iNOS	5′CTTGCCCCTGGAAGTTTCTC3′	5′GCAAGTGAAATCCGATGTGG3′
Cox-2	5′CAGTTTATGTTGTCTGTCCA3′	5′CCAGCACTTCACCCATCAGT3′
IL-1*β*	5′GTGTCTTTCCCGTGGACCTT3′	5′TCGTTGCTTGGTTCTCCTTG3′
TNF-*α*	5′CATCTTCTCAAAATTCGAGTGACAA3′	5′TGGGAGTAGACAAGGTACAACCC3′
IL-6	5′GTTTTCTGCAAGTGCATCATCG3′	5′GGTTTCTGCAAGTGCATCATCG3′

Abbreviations: Cox-2, cyclooxygenase-2; IL-1*β*, interleukin-1*β*; IL-6, interleukin-6; iNOS, inducible NO synthase; qPCR, quantitative real-time PCR; TNF-*α*, tumor necrosis factor-alpha.

## Data Availability

The datasets generated during and/or analyzed during the current study are not publicly available but are available from the corresponding author on reasonable request.

## References

[1] Hou Y., Dan X., Babbar M. (2019). Ageing as a Risk Factor for Neurodegenerative Disease. *Nature Reviews Neurology*.

[2] Peng C., Trojanowski J. Q., Lee V. M.-Y. (2020). Protein Transmission in Neurodegenerative Disease. *Nature Reviews Neurology*.

[3] Leng F., Edison P. (2021). Neuroinflammation and Microglial Activation in Alzheimer Disease: Where Do We Go From Here?. *Nature Reviews Neurology*.

[4] DiSabato D. J., Quan N., Godbout J. P. (2016). Neuroinflammation: The Devil Is in the Details. *Journal of Neurochemistry*.

[5] Voet S., Srinivasan S., Lamkanfi M., van Loo G. (2019). Inflammasomes in Neuroinflammatory and Neurodegenerative Diseases. *EMBO Molecular Medicine*.

[6] Olajide O. A., Kumar A., Velagapudi R., Okorji U. P., Fiebich B. L. (2014). Punicalagin Inhibits Neuroinflammation in LPS-Activated Rat Primary Microglia. *Molecular Nutrition & Food Research*.

[7] Eumkeb G., Sakdarat S., Siriwong S. (2010). Reversing *β*-Lactam Antibiotic Resistance of *Staphylococcus aureus* With Galangin From *Alpinia officinarum* Hance and Synergism With Ceftazidime. *Phytomedicine*.

[8] Saliba S. W., Jauch H., Gargouri B. (2018). Anti-Neuroinflammatory Effects of GPR55 Antagonists in LPS-Activated Primary Microglial Cells. *Journal of Neuroinflammation*.

[9] Das A., Kim S. H., Arifuzzaman S. (2016). Transcriptome Sequencing Reveals that LPS-Triggered Transcriptional Responses in Established Microglia BV2 Cell Lines Are Poorly Representative of Primary Microglia. *Journal of Neuroinflammation*.

[10] Kheyrollah M., Sabouni F., Farhadpour M., Haghbeen K. (2020). Neuroprotective Effect of *Lithospermum officinale* Callus Extract on Inflamed Primary Microglial Cells. *Current Pharmaceutical Biotechnology*.

[11] Thomi G., Surbek D., Haesler V., Joerger-Messerli M., Schoeberlein A. (2019). Exosomes Derived From Umbilical Cord Mesenchymal Stem Cells Reduce Microglia-Mediated Neuroinflammation in Perinatal Brain Injury. *Stem Cell Research & Therapy*.

[12] Justo A. F. O., Suemoto C. K. (2022). The Modulation of Neuroinflammation by Inducible Nitric Oxide Synthase. *Journal of Cell Communication and Signaling*.

[13] Vijitruth R., Liu M., Choi D.-Y., Nguyen X. V., Hunter R. L., Bing G. (2006). Cyclooxygenase-2 Mediates Microglial Activation and Secondary Dopaminergic Cell Death in the Mouse MPTP Model of Parkinson’s Disease. *Journal of Neuroinflammation*.

[14] Raffaele S., Lombardi M., Verderio C., Fumagalli M. (2020). TNF Production and Release from Microglia via Extracellular Vesicles: Impact on Brain Functions. *Cells*.

[15] Erta M., Quintana A., Hidalgo J. (2012). Interleukin-6, A Major Cytokine in the Central Nervous System. *International Journal of Biological Sciences*.

[16] Mendiola A. S., Cardona A. E. (2018). The IL-1*β* Phenomena in Neuroinflammatory Diseases. *Journal of Neural Transmission*.

[17] Kang G., Kong P. J., Yuh Y. J. (2004). Curcumin Suppresses Lipopolysaccharide-Induced Cyclooxygenase-2 Expression by Inhibiting Activator Protein 1 and Nuclear Factor *κ*B Bindings in BV2 Microglial Cells. *Journal of Pharmacological Sciences*.

[18] Yu Y., Shen Q., Lai Y. (2018). Anti-Inflammatory Effects of Curcumin in Microglial Cells. *Frontiers in Pharmacology*.

[19] Hesam S., Khoshkholgh-Sima B., Pourbadie H. G., Babapour V., Zendedel M., Sayyah M. (2018). Monophosphoryl Lipid A and Pam3Cys Prevent the Increase in Seizure Susceptibility and Epileptogenesis in Rats Undergoing Traumatic Brain Injury. *Neurochemical Research*.

[20] Li F., Xu L., Yaoying M., Yue G., Ting Y., Chao H. (2022). Monophosphoryl Lipid a Tolerance Against Chronic Stress-Induced Depression-Like Behaviors in Mice. *International Journal of Neuropsychopharmacology*.

[21] Pourbadie H. G., Sayyah M., Khoshkholgh-Sima B. (2018). Early Minor Stimulation of Microglial TLR2 and TLR4 Receptors Attenuates Alzheimer’s Disease-Related Cognitive Deficit in Rats: Behavioral, Molecular, and Electrophysiological Evidence. *Neurobiology of Aging*.

[22] Hosseini S. M., Pourbadie H. G., Sayyah M., Zibaii M. I., Naderi N. (2018). Neuroprotective Effect of Monophosphoryl Lipid A, A Detoxified Lipid A Derivative, in Photothrombotic Model of Unilateral Selective Hippocampal Ischemia in Rat. *Behavioural Brain Research*.

[23] Timmerman R., Burm S. M., Bajramovic J. J. (2018). An Overview of in Vitro Methods to Study Microglia. *Frontiers in Cellular Neuroscience*.

[24] Green L. C., Wagner D. A., Glogowski J., Skipper P. L., Wishnok J. S., Tannenbaum S. R. (1982). Analysis of Nitrate, Nitrite, and [15N] Nitrate in Biological Fluids. *Analytical Biochemistry*.

[25] Berti R., Williams A. J., Moffett J. R. (2002). Quantitative Real-Time RT—PCR Analysis of Inflammatory Gene Expression Associated With Ischemia—Reperfusion Brain Injury. *Journal of Cerebral Blood Flow & Metabolism*.

[26] Cai B., Seong K.-J., Bae S.-W., Chun C., Kim W.-J., Jung J.-Y. (2018). A Synthetic Diosgenin Primary Amine Derivative Attenuates LPS-Stimulated Inflammation via Inhibition of NF-*κ*B and JNK MAPK Signaling in Microglial BV2 Cells. *International Immunopharmacology*.

[27] Xiong Y., Mahmood A., Chopp M. (2018). Current Understanding of Neuroinflammation After Traumatic Brain Injury and Cell-Based Therapeutic Opportunities. *Chinese Journal of Traumatology*.

[28] Grigoriadis N., Van Pesch V. (2015). A Basic Overview of Multiple Sclerosis Immunopathology. *European Journal of Neurology*.

[29] Kwon H. S., Koh S.-H. (2020). Neuroinflammation in Neurodegenerative Disorders: The Roles of Microglia and Astrocytes. *Translational Neurodegeneration*.

[30] Lively S., Schlichter L. C. (2018). Microglia Responses to Pro-Inflammatory Stimuli (LPS, IFN*γ*+TNF*α*) and Reprogramming by Resolving Cytokines (IL-4, IL-10). *Frontiers in Cellular Neuroscience*.

[31] Zhang G., He J., Xie X., Yu C. (2012). LPS-Induced iNOS Expression in N9 Microglial Cells Is Suppressed by Geniposide via ERK, p38 and Nuclear Factor-*κ*B Signaling Pathways. *International Journal of Molecular Medicine*.

[32] González-Scarano F., Baltuch G. (1999). Microglia as Mediators of Inflammatory Degenerative Diseases. *Annual Review of Neuroscience*.

[33] Karlstetter M., Lippe E., Walczak Y. (2011). Curcumin Is a Potent Modulator of Microglial Gene Expression and Migration. *Journal of Neuroinflammation*.

[34] Guo L., Xing Y., Pan R. (2013). Curcumin Protects Microglia and Primary Rat Cortical Neurons against HIV-1 gp120-Mediated Inflammation and Apoptosis. *PLoS ONE*.

[35] He W., Yuan K., Ji B., Han Y., Li J. (2020). Protective Effects of Curcumin Against Neuroinflammation Induced by A*β*25–35 in Primary Rat Microglia: Modulation of High-Mobility Group Box 1, Toll-Like Receptor 4 and Receptor for Advanced Glycation End Products Expression. *Annals of Translational Medicine*.

[36] Sampson T. R., Debelius J. W., Thron T. (2016). Gut Microbiota Regulate Motor Deficits and Neuroinflammation in a Model of Parkinson’s Disease. *Cell*.

[37] Feng L., Luo G., Li Y. (2023). Curcumin-Dependent Phenotypic Transformation of Microglia Mediates Resistance to Pseudorabies-Induced Encephalitis. *Veterinary Research*.

[38] Michaud J. P., Hallé M., Lampron A. (2013). Toll-Like Receptor 4 Stimulation With the Detoxified Ligand Monophosphoryl Lipid A Improves Alzheimer’s Disease-Related Pathology. *Proceedings of the National Academy of Sciences*.

[39] Sorrenti V., Contarini G., Sut S. (2018). Curcumin Prevents Acute Neuroinflammation and Long-Term Memory Impairment Induced by Systemic Lipopolysaccharide in Mice. *Frontiers in Pharmacology*.

[40] Akaishi T., Abe K. (2018). CNB-001, a Synthetic Pyrazole Derivative of Curcumin, Suppresses Lipopolysaccharide-Induced Nitric Oxide Production through the Inhibition of NF-*κ*B and p38 MAPK Pathways in Microglia. *European Journal of Pharmacology*.

[41] Solaro R., Chiellini F., Battisti A. (2010). Targeted Delivery of Protein Drugs by Nanocarriers. *Materials*.

[42] Ismaili J., Rennesson J., Aksoy E. (2002). Monophosphoryl Lipid A Activates Both Human Dendritic Cells and T Cells. *The Journal of Immunology*.

[43] García-González P. A., Schinnerling K., Sepúlveda-Gutiérrez A. (2017). Dexamethasone and Monophosphoryl Lipid a Induce a Distinctive Profile on Monocyte-Derived Dendritic Cells through Transcriptional Modulation of Genes Associated With Essential Processes of the Immune Response. *Frontiers in Immunology*.

[44] Zhang J., Zheng Y., Luo Y., Du Y., Zhang X., Fu J. (2019). Curcumin Inhibits LPS-Induced Neuroinflammation by Promoting Microglial M2 Polarization via TREM2/TLR4/NF-*κ*B Pathways in BV2 Cells. *Molecular Immunology*.

[45] Cianciulli A., Calvello R., Porro C., Trotta T., Salvatore R., Panaro M. A. (2016). PI3k/Akt Signalling Pathway Plays a Crucial Role in the Anti-Inflammatory Effects of Curcumin in LPS-Activated Microglia. *International Immunopharmacology*.

